# Real-time activity and fall detection using transformer-based deep learning models for elderly care applications

**DOI:** 10.1136/bmjhci-2025-101439

**Published:** 2025-09-17

**Authors:** Raja Omman Zafar, Farhan Zafar

**Affiliations:** 1 Dalarna University—Campus Borlange, Borlänge, Sweden; 2 Department of Computer Science, Capital University of Science and Technology, Islamabad, Islamabad Capital Territory, Pakistan

**Keywords:** Artificial intelligence, BMJ Health Informatics, Data Science, Deep Learning, Delivery of Health Care

## Abstract

**Objective:**

This study aims to develop a transformer-based deep learning model for real-time activity recognition and fall detection, addressing the limitations of existing methods in terms of accuracy and real-time applicability.

**Methods:**

The proposed system uses sliding window segmentation technique to process wearable sensor data, including accelerometer, gyroscope and orientation signals. The transformer encoder models temporal dependencies through a self-attention mechanism, enabling the extraction of global and local temporal patterns. The performance of the model is evaluated on an updated version of the MobiAct data set, which includes over 14 million sensor records collected from 66 participants and 16 activities, including four types of falls and multiple scenario-based activities of daily living.

**Result:**

The transformer model achieved an accuracy of over 98% and demonstrated excellent precision and recall for difficult fall categories such as forward-lying and sideward-lying. Comparative analysis shows that transformers outperform convolutional neural networks long short-term memory (CNN-LSTM) and temporal convolutional networks in terms of classification metrics, confusion matrix results and training stability.

**Discussion:**

The results highlight the effectiveness of the transformer model in capturing complex temporal dependencies, addressing key challenges such as misclassification and false positives. Compared with traditional models, its parallel processing capabilities improve real-time deployment efficiency.

**Conclusion:**

This research establishes transformer-based models as powerful solutions for activity recognition and fall detection, providing reliable applications for elderly care and fall prevention. Future work will focus on optimising edge devices and validating on real-world data sets.

**WHAT IS ALREADY KNOWN ON THIS TOPIC**

- Existing activity recognition and fall detection systems, such as convolutional neural networks, long short-term memory and temporal convolutional network, achieve excellent accuracy but face challenges in real-time applications, including distinguishing complex fall types and handling false positives. Transformer’s self-attention mechanism shows promise in modelling temporal dependencies, but its application in wearable sensor-based fall detection remains underexplored.

**WHAT THIS STUDY ADDS**

- This study shows that transformer-based models can outperform traditional architectures in terms of accuracy and precision in fall detection, especially for difficult fall categories such as forward-lying and sideward-lying. The model’s ability to process sequences in parallel improves real-time applicability, providing a more robust solution for activity and fall identification.

**HOW THIS STUDY MIGHT AFFECT RESEARCH, PRACTICE OR POLICY**

- This study highlights the potential of transformer-based models for real-time health monitoring, especially for elderly care. These findings could inform the development of scalable and effective wearable systems for fall prevention, thereby reducing healthcare costs and improving patient outcomes. Future research can leverage this architecture to enable state-of-the-art device implementation and validation on real-world falls induced in a controlled laboratory environment.

## Introduction

 Falls are a leading cause of injury and hospitalisation among older adults worldwide, resulting in millions of emergency room visits each year.^1^ As the global population ages, there is an increasing need for reliable systems to monitor daily activities and detect falls in real-time.^2^ Accurate fall detection can mitigate injuries through rapid medical intervention, while activity recognition can provide valuable insights into behaviour and overall health.^3^ Advances in wearable sensor technology, including accelerometers, gyroscopes and orientation sensors, now enable continuous monitoring of high-frequency body activity.^4^ These sensors, often integrated into wearable devices, classify activity of daily living (ADL) such as walking (WAL), sitting and standing (STD), and detect falls such as leaning forward or lying on one’s side. However, existing systems face challenges in achieving high accuracy for underrepresented fall categories and meeting the computational demands of real-time processing.^5^,^7^

Human activity recognition (HAR) has attracted widespread research interest due to its applications in health monitoring, smart cities and elderly care. Many methods have been explored to improve HAR performance, including supervised learning methods such as random forests and k-nearest neighbours and hybrid models such as convolutional neural networks long short-term memory (CNN-LSTM), which extract spatial and temporal features of sensor data.^4 8 9^ Privacy-preserving methods, such as radar-based HAR using micro-Doppler signatures, have also attracted attention due to their contactless and non-intrusive nature.^10^ Integrated techniques, such as combining decision trees with AdaBoost, have further demonstrated improvements in recognition accuracy on benchmark data sets.^6^ Emerging deep learning architectures, including hybrid CNN-LSTM and transformer-based models, show promise in capturing complex spatial and temporal dependencies in activity data, making them highly effective for HAR tasks.^9 11^ However, the overlap between daily life events and fall events often leads to misclassification, requiring more robust methods.^12^

When it comes to fall detection, wearable systems and IoT-based (Internet-of-Things) technologies have played a central role in advancements. Accelerometer and gyroscope data are used with machine learning models such as random forests and Kalman filters to detect falls in real time, providing critical time for interventions.^12 13^ Context-aware systems, such as smart pads, use sensor data for real-time monitoring with high sensitivity and specificity.^14^ IoT-enabled architectures that combine edge and cloud computing have demonstrated their scalability and efficiency, using decision trees to efficiently transmit and process data in large-scale deployments.^7^ Despite these advances, current systems still suffer from high false positive rates and computational inefficiency, especially when dealing with complex falls in real-world scenarios.^6 15^

To address these limitations, we propose a transformer-based deep learning model designed for real-time activity recognition and fall detection. Leveraging transformers’ self-attention mechanism, the model excels at capturing local and global temporal dependencies in wearable sensor data. By employing techniques such as sliding window segmentation, majority voting and predictive smoothing, the model improves classification stability and reduces false positives. An in-depth evaluation of the MobiAct data set demonstrates the state-of-the-art performance of the system, particularly in detecting difficult fall categories such as forward-lying (FOL) and sideward-lying (SDL). By comparing the transformer model with traditional models such as CNN-LSTM and temporal convolutional network (TCN), we highlight its robustness and potential for practical applications in elderly care.^7 9 11^

## Methods

### Data set and data preprocessing

The study used the MobiAct data set, a well-known benchmark for activity recognition and fall detection research.^16^ The data set contains data from wearable sensors, including accelerometers, gyroscopes and orientation signals recorded during various activities. These activities fall into two main categories: ADL and falls, as shown in table 1. To prepare the data for model training, several preprocessing steps are performed. Sensor data is normalised to ensure uniform scaling and eliminate potential bias due to different signal ranges. Additionally, a fourth-order low-pass Butterworth filter with a 3 Hz cut-off frequency was applied to the accelerometer channels to remove high-frequency noise and improve signal quality prior to segmentation. The data set was segmented using a sliding window method, with each window consisting of 100 samples with a stride of 50. The most common tabs in each window are designated as representative tabs. This approach allows the model to efficiently learn temporal patterns across overlapping windows. Furthermore, class imbalance in the data set was corrected using the Synthetic Minority Oversampling Technique, generating synthetic samples for underrepresented fall events, ensuring the data set remains consistently balanced during training.

**Table 1:** Different types of codes and their activities

Code	Activity	Duration	Description
STD	Standing	5 min	Standing with subtle movements
WAL	Walking	5 min	Normal walking
JOG	Jogging	30 s	Jogging
JUM	Jumping	30 s	Continuous jumping
STU	Stairs up	10 s	Stairs up (10 stairs)
STN	Stairs down	10 s	Stairs down (10 stairs)
SCH	Stand to sit	1 min	Transition from standing to sitting
SIT	Sitting on a chair	1 min	Sitting on a chair with subtle movements
CHU	Sit to stand	6 s	Transition from sitting to standing
CSI	Car-step in	6 s	Step in a car
CSO	Car-step out	6 s	Step out of a car
LYI	Lying	6 s	Activity taken from lying period after a fall
FOL	Forward-lying	10 s	Fall forward from standing, use of hands
FKL	Front-knees-lying	10 s	Fall forward from standing, first impact on knees
BSC	Back-sitting-chair	10 s	Fall backward while trying to sit on a chair
SDL	Sideward-lying	10 s	Fall sidewards from standing, bending legs

### Model architecture

**Figure 1:**
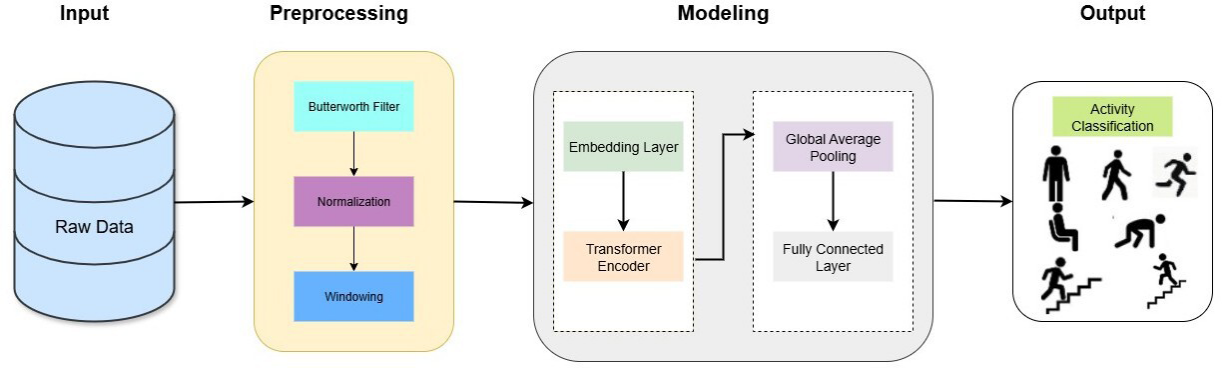
Proposed transformer-based deep learning architecture.

The proposed transformer-based model as shown in figure 1 is designed to effectively handle temporal dependencies in wearable sensor data, making it particularly suitable for activity recognition and fall detection. The model starts with an input embedding layer, which maps raw sensor data to a higher-dimensional latent space, thereby improving its representation capacity. At the core of the architecture is the transformer encoder, which uses a self-attention mechanism to capture local and global dependencies in the input sequences. The encoder leverages a multihead self-attention mechanism, allowing the model to attend to different temporal segments in parallel. This improves the model’s ability to capture various temporal dependencies between different sensor modalities. In this implementation, acceleration, gyroscope and orientation signals are processed uniformly and transmitted through shared encoders and embedding. The architecture is encoder-only, as the task involves sequence classification rather than sequence generation. After processing through the transformer layer, the output is aggregated using global average pooling, which compresses the temporal features into a fixed-size representation. Finally, a fully connected layer maps the aggregated features to activity categories, including ADLs and falls. This architecture ensures robust learning of temporal patterns while maintaining computational efficiency, which is critical for real-time applications.

### Evaluation metrics

The performance of the model is evaluated using various metrics to assess its accuracy and robustness. Precision is calculated to measure the overall accuracy of predictions, while precision and recall evaluate a model’s ability to correctly classify specific activity categories and identify all relevant instances of those categories, respectively. F1-Score is the harmonic average of precision and recall and is used as a balanced metric for performance evaluation. In addition to these metrics, a confusion matrix was generated to visualise the classification performance for all activity categories. The matrix provides information on strengths and weaknesses in specific areas, such as correctly identifying fall events (FOL, front-knees-lying (FKL), back-sitting-chair (BSC), SDL) and differentiating them from ADL.

### Signal visualisation for fall activities

To better understand fall activity, the sensor signals corresponding to different falls were visualised (online supplemental figure S1). Accelerometer and gyroscope data were plotted for each fall type (FOL, FKL, BSC, SDL), highlighting the different patterns and characteristics unique to each activity. For example, FOL position often exhibits sudden peaks in the accelerometer signal in the vertical axis, while the SDL position exhibits clear changes in lateral acceleration. These visualisations help understand the temporal and spatial dynamics of fall activity, providing an explanatory layer for model predictions. They also revealed potential difficulties in distinguishing certain types of falls, such as FKL and BSC, which have similar signalling patterns.

## Results

### Classification performance

**Figure 2:**
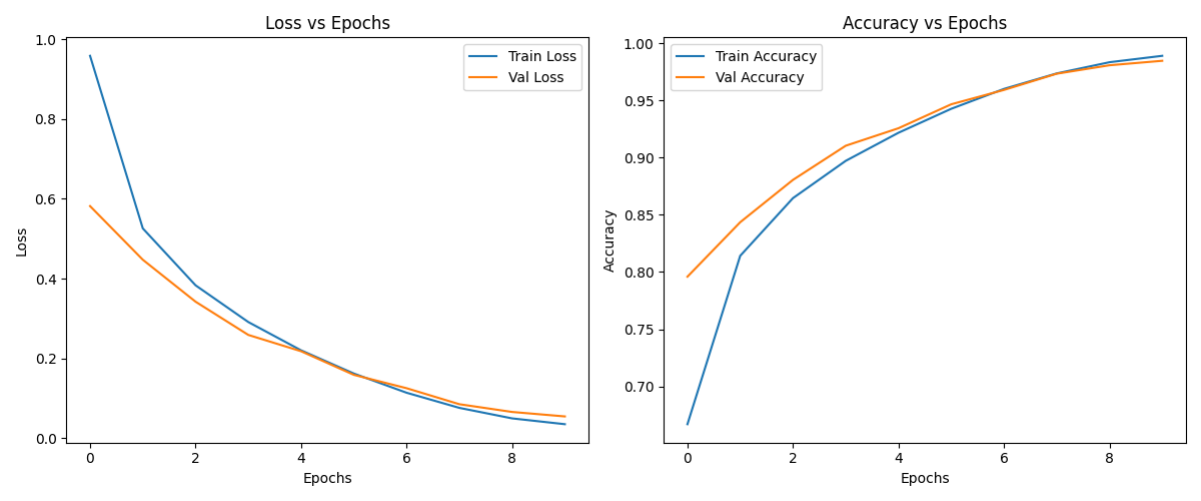
Transformer-based model training and testing graphs. Val, validation.

**Figure 3:**
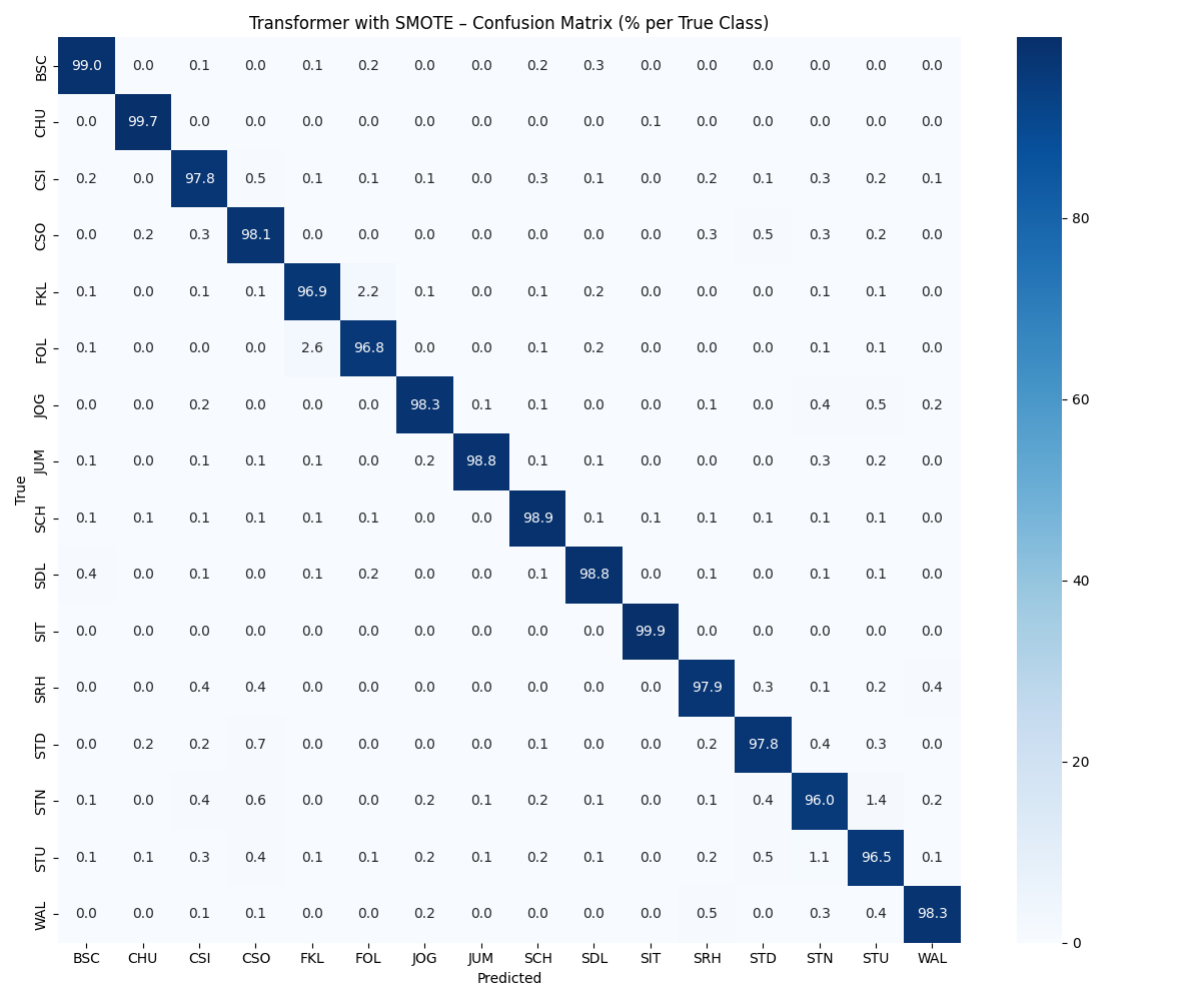
Transformer-based model confusion matrix result. BSC, back-sitting-chair; CHU, sit to stand; CSI, car-step in; CSO, car-step out; FKL, front-knees-lying; FOL, forward-lying; JOG, jogging; JUM, jumping; SCH, stand to sit; SDL, sideward-lying; SIT, sitting on chair; SMOTE, Synthetic Minority Oversampling Technique; SRH, Scenario of Returning at Home; STD, standing; STN, stairs down; STU, stairs up; WAL, walking.

Transformer model demonstrated exceptional performance on the MobiAct data set, effectively distinguishing between ADL and fall activities. The training and testing accuracy graphs (figure 2) illustrate the model’s consistent improvement and stability across epochs, with the testing accuracy exceeding 98%. This high accuracy underscores the model’s ability to generalise well to unseen data. The model excelled in recognising activities such as WAL, STD and jogging, which exhibit distinct temporal patterns, but faced challenges with more nuanced fall categories like car-step in (CSI) and car-step out (CSO) due to their overlap with daily activities. The confusion matrix (figure 3) further highlights the model’s ability to minimise misclassification across activity categories, particularly for underrepresented fall activities, showcasing its robustness in handling complex patterns within wearable sensor data.

### Comparative analysis

**Figure 4:**
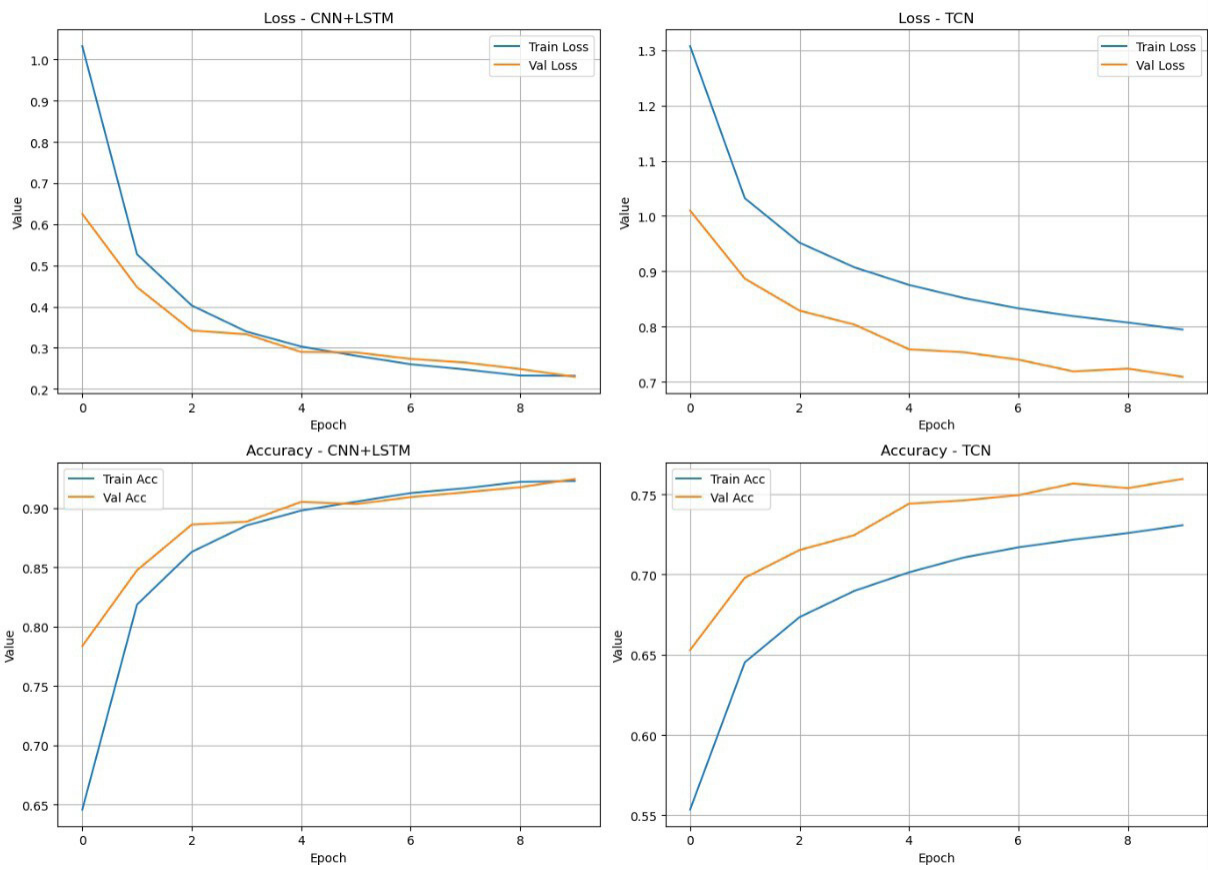
CNN-LSTM and TCN model training and testing graphs. Acc, accuracy; CNN-LSTM, convolutional neural networks long short-term memory; TCN, temporal convolutional network; Val, validation.

The performance of the transformer model is benchmarked against CNN-LSTM and TCN using the MobiAct data set. The accuracy and loss plots (figure 4) illustrate the training and validation performance over epochs. CNN-LSTM exhibits rapid convergence and achieves a validation accuracy of over 92%, demonstrating its strength in modelling temporal patterns when sufficient training epochs are allowed. TCN shows a slower learning curve, plateauing at around 76% validation accuracy. While TCN benefits from stable training dynamics and smoother loss reduction, it underperforms compared with CNN-LSTM in overall classification accuracy. However, both models fall short of the performance achieved by the transformer, which not only maintains higher accuracy but also generalises better across both ADLs and fall classes. These trends are further confirmed by the confusion matrices (online supplemental figure S2), where CNN-LSTM shows reduced misclassification across fall categories, but still exhibits limitations in edge cases such as SDL and FKL, while TCN struggles with broader overlap between fall and non-fall activities.

### Confusion matrix analysis

The confusion matrix provides valuable insights into the transformer model’s performance in differentiating falls from ADL. Dividing activities into two categories, falls and ADLs, showed high true positive rates for both groups, demonstrating the model’s effectiveness in identifying and classifying these broad activity categories. However, a closer look at the individual activity sessions in these groups revealed areas for improvement. For example, certain edge activities, such as CSI and CSO, often lead to false positives or misclassifications. These activities share overlapping signal patterns with other ADLs or falls, making it particularly difficult for models to accurately distinguish between them. For example, FKL is sometimes misclassified as STD due to similar acceleration profiles, while CSO is sometimes confused with falls that involve gradual deceleration, such as SDL.

## Discussion

The results of this study demonstrate the potential of transformer-based deep learning models for accurate and efficient real-time activity recognition and fall detection. By leveraging the temporal processing capabilities of the self-attention mechanism, the proposed model outperforms traditional methods such as CNN-LSTM and TCN in several key areas such as classification accuracy, handling of imbalanced categories and real-time performance. However, the results also highlight challenges and opportunities for further improvements.

### Performance of transformer models

Transformer model achieves an overall classification accuracy of over 98%, with particularly high precision and recall for ADL. This performance is attributed to the model’s ability to capture local and global temporal dependencies, allowing it to distinguish subtle patterns in sensor data. For example, the model effectively differentiates between falling activities such as FOL and FKL, which exhibit different signalling patterns. The use of global average pooling in the architecture further reduces complexity and ensures that the system can run smoothly in a real-time environment. The choice of a window size of 100 (equivalent to 2 s at 50 Hz) is supported by previous HAR literature and our own thorough analysis (online supplemental figure S3), which shows that this window size achieves a high F1-score while striking a good balance between temporal context and computational cost.^17^

### Model comparison

Comparative analysis highlights the advantages of the transformer model over CNN-LSTM and TCN. Although CNN-LSTM performs well in ADL, its sequential nature limits its effectiveness in real-time applications. In contrast, TCN is good at capturing long-term dependencies but struggles with complex transitions between fall activities and ADLs. Transformer models address these limitations by combining parallel processing with powerful self-attention mechanisms.

### Challenges with under-represented fall classes

Despite its strong performance on the MobiAct data set, the transformer model faced challenges in some under-represented fall courses, such as CSI and CSO. One potential reason for the misclassification between CSI, SCO and SDL is the limited discriminative power of signals captured by a single sensor (eg, waist or pocket). These activities involve similar vertical and transitional movements, which may be indistinguishable in torso-based IMU (inertial measurement unit) data. This issue highlights the need for additional strategies to increase model sensitivity to subtle differences in fall activity. To address this issue, techniques such as class-weighted loss functions and synthetic data augmentation can be used during training.^18^ Although these methods improve performance, additional improvements, such as placing sensors on the feet or legs, can capture gait dynamics and height changes, while placing sensors on the pelvis or hips can better reflect body transitions. Combining these position-aware data can improve the model’s ability to distinguish these subtle activities.

### Applications in elderly care

The high accuracy and real-time capabilities of the transformer-based system make it a promising solution for deployment in wearable devices for elderly care. By continuously monitoring activity and detecting falls with minimal false alarms, the system can improve the safety and quality of life of older adults. For example, it can provide caregivers with timely alerts and detailed activity reports to intervene faster in the event of a fall. However, practical deployment of such systems requires consideration of other factors such as energy efficiency, model optimisation of edge devices and user adaptability.^19^ A lightweight transformer architecture and hardware-specific optimisations such as quantisation and pruning can make the system more suitable for portable applications.

### Limitations and future directions

Although the proposed system exhibits strong performance, there are still some limitations. The current evaluation is based on the MobiAct data set, which significantly comprises comprehensive data sets (66 participants and over 14 million sensor records), still relies on simulated falls and controlled experimental settings. As such, further testing is needed to confirm the model’s robustness under real-world conditions and across different populations, including older adults. Furthermore, although postprocessing techniques do improve prediction stability, they introduce a slight delay that may be critical for time-sensitive applications such as fall prevention. The system’s current focus on identifying predefined activities and falls also limits its applicability; expanding the activity repertoire or enabling unsupervised detection of novel activities could address this issue. Future work could explore modality-specific ensembles or attention heads to better capture the different dynamics of each sensor type. Future work will also focus on evaluating the model on other data sets, such as SisFall, which contains a wider range of fall scenarios and sensor locations. This will allow us to evaluate the generalisability of the model to different populations and environments.^20^ In addition, integrating reinforcement learning techniques can enable the model to dynamically adapt to new activities and changing environments, improving its overall flexibility and real-world applicability.^21^

## Conclusion

This study demonstrates the potential of transformer-based deep learning models for real-time activity recognition and fall detection using wearable sensor data. By leveraging the self-attention mechanism, the proposed model effectively captures complex temporal patterns, thereby achieving state-of-the-art accuracy on the MobiAct data set. The model’s overall classification accuracy exceeds 98%, outperforming traditional architectures such as CNN-LSTM and TCN, especially in differentiating difficult fall categories and ADL aspect. The system’s applicability to elderly care is particularly promising. By continuously monitoring daily activities and providing timely fall alerts, the system can significantly improve the safety and quality of life of older adults. Its real-time functionality makes it suitable for deployment in wearable devices, allowing caregivers to quickly respond to emergencies and track activity patterns for long-term health monitoring.

## Supplementary material

10.1136/bmjhci-2025-101439 online supplemental file 1

## Data Availability

Data are available upon reasonable request. Data may be obtained from a third party and are not publicly available.
