# Supplementary material for: Real-time activity and fall detection using transformer-based deep learning models for elderly care applications
Source: BMJ Health Care Inform. 2025 Sep 17;32(1):e101439. doi: 10.1136/bmjhci-2025-101439 (PMC12458858; doi:10.1136/bmjhci-2025-101439)

# Supplementary Material

Title: **Real-Time Activity and Fall Detection Using Transformer-Based Deep Learning Models for Elderly Care Applications**

Supplemental Figure S1. Sensor signals visualization corresponding to different falls


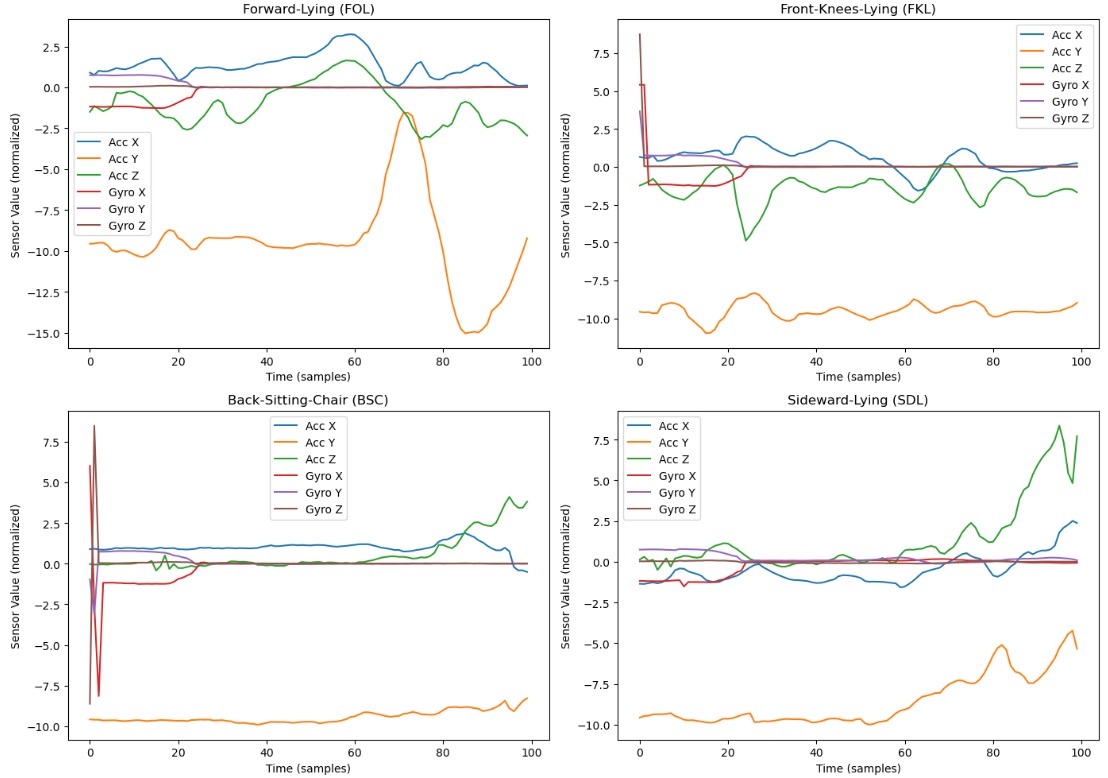


Supplemental Figure S2. CNN-LSTM and TCN confusion matrix result


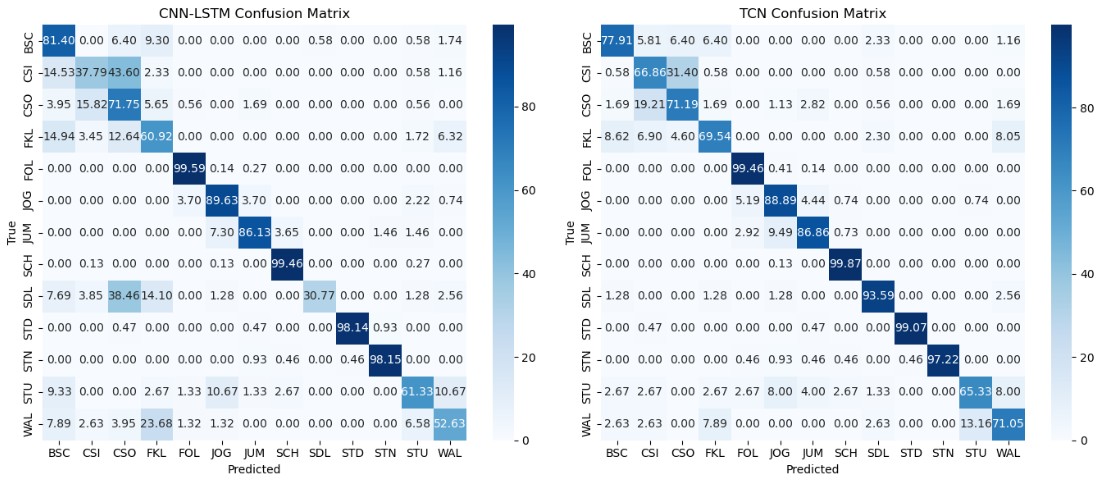


Supplemental Figure S3. Training and validation performance across different window size


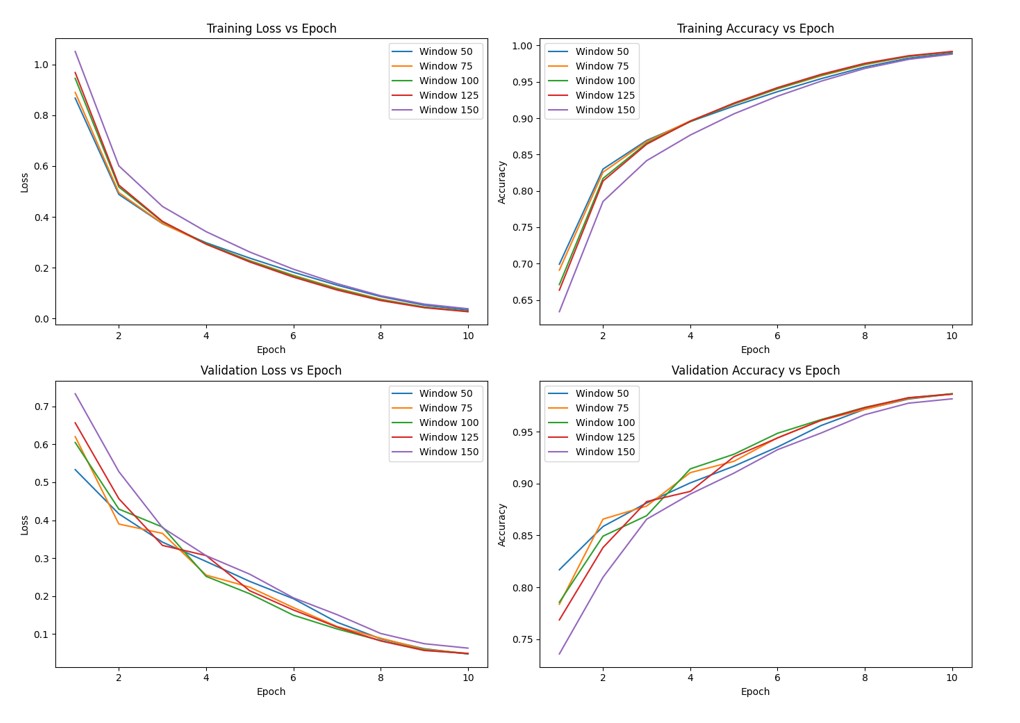

Supplement: online supplemental file 1 [file bmjhci-32-1-s001.docx]
